# Falciparum but not vivax malaria increases the risk of hypertensive disorders of pregnancy in women followed prospectively from the first trimester

**DOI:** 10.1186/s12916-021-01960-3

**Published:** 2021-04-27

**Authors:** Whitney E. Harrington, Kerryn A. Moore, Aung Myat Min, Mary Ellen Gilder, Nay Win Tun, Moo Kho Paw, Jacher Wiladphaingern, Stephane Proux, Kesinee Chotivanich, Marcus J. Rijken, Nicholas J. White, François Nosten, Rose McGready

**Affiliations:** 1 Shoklo Malaria Research Unit, Mahidol-Oxford Tropical Medicine Research Unit, Faculty of Tropical Medicine, Mahidol University, Mae Sot, Thailand; 2 Department of Pediatrics, University of Washington, Seattle, WA USA; 3 Seattle Children’s Research Institute, Seattle, WA USA; 4 London School of Hygiene and Tropical Medicine, London, UK; 5 Murdoch Children’s Research Institute, Melbourne, Australia; 6 Faculty of Tropical Medicine, Mahidol University, Bangkok, Thailand; 7 Utrecht University Medical Centre, Utrecht, the Netherlands; 8 Julius Centre Global Health, Utrecht, the Netherlands; 9 Centre for Tropical Medicine and Global Health, Nuffield Department of Medicine, University of Oxford, Old Road Campus, Oxford, UK; 10 Mahidol-Oxford Tropical Medicine Research Unit, Faculty of Tropical Medicine, Mahidol University, Bangkok, Thailand

**Keywords:** First trimester, Falciparum malaria, Pre-eclampsia, Gestational hypertension, Vivax malaria

## Abstract

**Background:**

Malaria and hypertensive disorders of pregnancy (HDoP) affect millions of pregnancies worldwide, particularly those of young, first-time mothers. Small case-control studies suggest a positive association between falciparum malaria and risk of pre-eclampsia but large prospective analyses are lacking.

**Methods:**

We characterized the relationship between malaria in pregnancy and the development of HDoP in a large, prospectively followed cohort. Pregnant women living along the Thailand-Myanmar border, an area of low seasonal malaria transmission, were followed at antenatal clinics between 1986 and 2016. The relationships between falciparum and vivax malaria during pregnancy and the odds of gestational hypertension, pre-eclampsia, or eclampsia were examined using logistic regression amongst all women and then stratified by gravidity.

**Results:**

There were 23,262 singleton pregnancies in women who presented during the first trimester and were followed fortnightly. Falciparum malaria was associated with gestational hypertension amongst multigravidae (adjusted odds ratio (AOR) 2.59, 95%CI 1.59–4.23), whereas amongst primigravidae, it was associated with the combined outcome of pre-eclampsia/eclampsia (AOR 2.61, 95%CI 1.01–6.79). In contrast, there was no association between vivax malaria and HDoP.

**Conclusions:**

Falciparum but not vivax malaria during pregnancy is associated with hypertensive disorders of pregnancy.

## Background

Worldwide, hypertensive disorders of pregnancy (HDoP) are estimated to affect 3–10% of all pregnancies [1–3], with the greatest risk in the first pregnancies of young women [4]. The clinical presentation ranges from gestational hypertension to pre-eclampsia to life-threatening eclampsia [1]. It is unclear whether gestational hypertension and pre-eclampsia are separate entities or represent a spectrum of disease as they share some but not all risk factors and result in varying placental pathology, with findings consistent with placental ischemia specifically associated with pre-eclampsia [5]. In contrast, eclampsia, or maternal seizures, is considered to be the central nervous system-specific presentation of severe pre-eclampsia [6]. Maternal spiral arteries undergo extensive remodeling during a key window of 6 to 20 weeks’ gestation allowing for appropriate perfusion of the placenta [7]. Accumulating evidence suggests that pre-eclampsia is the product of abnormal spiral artery development leading to placental hypoxia, triggering the release of fetal-derived molecules that increase maternal blood pressure and perfusion of the placenta but result in diffuse endothelial dysfunction in the mother [4].

Primigravidae are also particularly susceptible to *Plasmodium falciparum* malaria in pregnancy with a peak of detection at 13–18 weeks’ gestation in higher transmission settings [8], corresponding to the key period of fetal trophoblast invasion and maternal spiral artery transformation [9]. Falciparum malaria early in pregnancy has been associated with abnormal placental vascular development [10], and falciparum-infected erythrocytes may sequester in the placental intervillous spaces, a condition known as placental malaria, leading to inflammatory infiltrates and low birth weight [11]. Peripheral falciparum malaria in the absence of placental infection has also been associated with poor pregnancy and birth outcomes including miscarriage [12]; pre-term delivery [13]; intrauterine growth retardation [14], small for gestational age [13], and low birth weight [15]; stillbirth [16]; and neonatal death [16]. In contrast, *Plasmodium vivax* malaria does not display a placental tropism, but in the present study area, has also been associated with poor pregnancy outcomes similar to the effect of falciparum malaria [12, 13, 16].

In West Africa, an increase in pre-eclampsia [17] and maternal deaths due to eclampsia has been noted during [18, 19] or immediately after [20] the malaria transmission season. Small studies have linked placental falciparum malaria infection, particularly in young primigravidae [21], with gestational hypertension [21, 22] and pre-eclampsia [17, 23]. Molecular mediators of pre-eclampsia have also been associated with falciparum malaria [21, 24]. Other studies, however, have not found an association between peripheral [25] or placental [26] falciparum malaria and pre-eclampsia. No studies have examined the association between vivax malaria or other human malarias and HDoP. We hypothesized that peripheral falciparum malaria, but not vivax malaria, would increase the risk of HDoP via its effect on placental function and that this effect would be most pronounced with infections occurring early in pregnancy during the key window of placental vascular development.

We therefore examined the association between falciparum and vivax malaria in pregnancy and HDoP along the Thailand-Myanmar border, an area of low seasonal malaria transmission, in a large cohort of women who attended antenatal clinics regularly from the first trimester of pregnancy.

## Methods

### Cohort and antenatal care

The Shoklo Malaria Research Unit (SMRU) has worked with displaced persons since 1986 and migrant populations since 1998. Clinics are situated along the Thailand-Myanmar border and offer free antenatal and birth care. Antenatal records of singleton births occurring at or after 28 weeks’ gestation between 1986 and 2016 were obtained. The analysis was restricted to women who began antenatal attendance in the first trimester (< 14 weeks’ gestation). Women were encouraged to attend an antenatal clinic (ANC) weekly when malaria transmission was high, and fortnightly as transmission decreased, and to attend at any time if ill. The woman’s age, parity, refugee status, number of consultations, weight at enrollment, weight gain during pregnancy, location and year of delivery, and birth outcome were recorded. Data on smoking in pregnancy were obtained between 1997 and 2016. Gestational age was assessed preferentially by ultrasound biometry (2001 to 2016) but if not available by Dubowitz Gestational Age Assessment (1992–2002) and finally by fundal height (1986–1994). As there are no safe and effective chemoprophylactics for pregnant women in settings of multidrug-resistant *P. falciparum*, malaria was detected actively by blood smears obtained at each ANC visit. Hematocrit was also assessed at each visit; moderate and severe anemia was defined as < 30% and < 20%, respectively. Blood pressure (BP) was measured by a manual sphygmomanometer with the woman recumbent. BP was monitored at least monthly, weekly in the last 4 weeks of pregnancy, and at every malaria episode. A high blood pressure reading was confirmed by repeating the reading after 30 min to confirm that the blood pressure remained elevated after the woman was allowed to relax. Proteinuria by urine dipstick was assessed if BP was elevated (systolic BP ≥ 140 and/or diastolic BP ≥ 90 mmHg).

### Clinical information

From the first ANC visit to delivery, vitamin supplements were provided: non-anemic women received iron (ferrous sulfate 200 mg daily), folic acid (5 mg weekly), and vitamin B1 (100 mg daily). Women with anemia received higher doses of ferrous sulfate (400 mg twice daily) and folic acid (5 mg daily) as well as vitamin C and vitamin B12 (both 100 mg twice daily).

### Clinical definitions

Throughout the many years of the study, a rigorous and consistent definition of the clinical syndromes was used.

#### Hypertension

Hypertension was two confirmed episodes of high blood pressure > 6 h apart with systolic BP ≥ 140 or diastolic BP ≥ 90.

#### Chronic hypertension

Chronic hypertension was hypertension by medical history or documented before 20 weeks’ gestation.

### Gestational hypertension

Gestational hypertension was new hypertension that occurred after 20 weeks’ gestation with no/trace of proteinuria.

### Pre-eclampsia

Pre-eclampsia was new onset hypertension that occurred after 20 weeks’ gestation with proteinuria (≥1+ on a dipstick), without alternative explanation. For women with a history of chronic hypertension, the onset of new proteinuria was after 20 weeks’ gestation.

### Eclampsia

Eclampsia was observed generalized convulsion (i.e., “fitting”) in a pregnant woman without other clear explanation, with a record of proteinuria or hypertension.

### Hypertensive disorder of pregnancy

Hypertensive disorder of pregnancy was a combined outcome of gestational hypertension, pre-eclampsia, or eclampsia.

### Malaria exposure

Malaria was defined as the presence of asexual parasites on a blood smear. *Plasmodium* species and parasitemia were determined by microscopy for all positive smears. All malaria infections were treated irrespective of symptoms; *P. falciparum* was treated with quinine or artemisinin-based combination therapy, and *P. vivax* was treated with chloroquine. The majority of treatments were supervised. Women with positive blood smears were followed with daily malaria smear until negative. Recurrence or reinfection after treatment completion and negative blood smear was considered possible from day 7 after the primary diagnosis.

### Statistical analysis

Each woman was considered in a single outcome category (no HDoP, gestational hypertension, pre-eclampsia, or eclampsia) according to the maximum signs and symptoms she experienced during the pregnancy. For example, if a woman first developed gestational hypertension but later developed proteinuria, she was considered as a final diagnosis of pre-eclampsia; if she went on to develop seizures, she was considered as a final diagnosis of eclampsia. For analyses of falciparum malaria, women with only *P. falciparum* detected during the pregnancy represented the exposed group, regardless of the number of infections, and women with no *Plasmodium* of any species detected served as the control group. The same approach was taken for the analysis of vivax malaria. Given the likely placental origin of HDoP, we restricted the analysis to women who experienced infection after 6 weeks of gestation. Logistic regression was used to estimate the association between falciparum and vivax malaria in pregnancy and each outcome separately. As eclampsia is considered to be a feature of severe pre-eclampsia and there were relatively few eclampsia outcomes, we additionally considered the composite outcome of pre-eclampsia or eclampsia. Models were adjusted for confounding variables: gravidity, maternal age, population (migrant versus refugee), first-trimester weight, pregnancy weight gain, chronic hypertension, year, and place of delivery. Women with pre-pregnancy hypertension were excluded from the model of gestational hypertension. First-trimester weight was used in place of body mass index (BMI) as height was available for a limited number of women (Additional file 1). To assess for confounding by smoking, a subset analysis was conducted amongst women for whom smoking data were available (Additional file 2). Because of prior reports of a gravidity-specific relationship between falciparum malaria and HDoP, accentuated amongst young women [21], we evaluated an interaction term between malaria and gravidity to generate the gravidity-specific effect for each outcome, as well as an interaction term between malaria, gravidity, and maternal age.

To determine if the association between malaria and HDoP was modified by the gestational age at first detection, we added an interaction term between malaria and the gestational age of the first detection as a continuous variable to the adjusted models. The assumption that the change in the association between malaria and the outcome across levels of gestational age was linear was tested visually by plotting the estimated log odds ratios within 4-week gestational age windows and statistically by including an interaction with gestational age as a quadratic term. We also included an interaction between malaria and gravidity as in the primary analysis, but we did not let the interaction between malaria and gestational age be modified by gravidity as there was no evidence that this interaction was different between primigravidae and multigravidae.

## Results

### Prevalence of malaria and hypertensive disorders of pregnancy

**Fig. 1:**
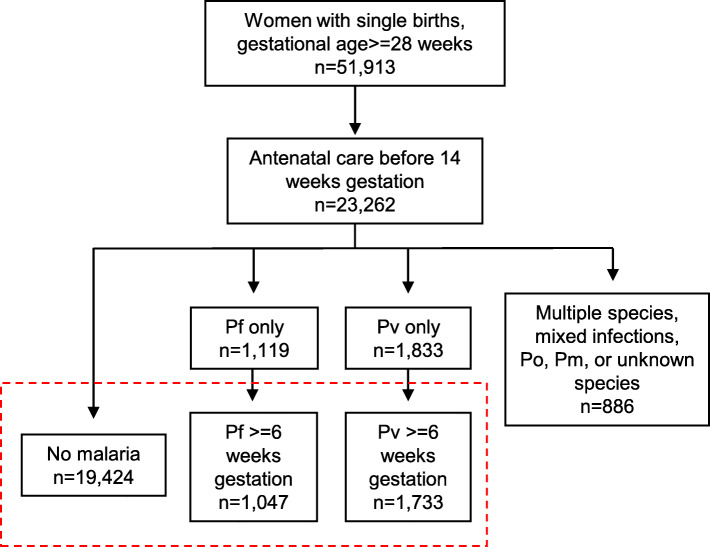
Distribution of *Plasmodium* infections within the cohort. Pf, *Plasmodium falciparum*; Pv, *Plasmodium vivax*; Po, *Plasmodium ovale*; Pm, *Plasmodium malaria*. The final cohort denoted in the red box included 22,204 women with single births after 28 weeks’ gestation who received antenatal care before 14 weeks of gestation: 19,424 women with no malaria during the pregnancy, 1047 with falciparum malaria after 6 weeks’ gestation, and 1733 with vivax malaria after 6 weeks’ gestation

Of 51,913 women who delivered at or after 28 weeks’ gestation between 1986 and 2016, 23,262 (45%) began antenatal care in their first trimester and had a singleton pregnancy (Fig. 1). Most women, 19,424 (83.5%), did not have malaria detected during their pregnancy. A total of 1047 women (4.5%) had falciparum malaria only with the first detection at or after 6 weeks’ gestation, and 1733 (7.5%) had vivax malaria only with the first detection at or after 6 weeks’ gestation; these three groups (22,204 total women) comprised the final cohort for analysis. An additional 1058 (4.5%) women had falciparum or vivax malaria before 6 weeks’ gestation, infections with multiple species, mixed infections, *P. ovale*, *P. malariae*, or infection with unknown species and were excluded from the analysis.

Women presented to antenatal care early in their pregnancy (median 9 weeks) and delivered with a median gestational age at delivery of 39 weeks (Table 1). The median gestational age at the first detection of falciparum malaria was 16 weeks (Additional file 3), and 759 (72.5%) women had a single episode (the maximum was 6 discrete episodes). The median gestational age at the first detection of vivax malaria was 16 weeks (Additional file 3), and 960 (55.4%) women had a single episode only (the maximum was 8 discrete episodes). Gestational age when malaria was first detected was inversely correlated with the number of episodes.

**Table 1:** Participant characteristics by *Plasmodium* exposure

Characteristic	No malaria	*P. falciparum*	*P. vivax*
Number	19,424	1047	1733
Maternal age, years, mean (SD)	26.2 (6.5)	25.2 (6.2)	25.4 (6.7)
Gravidity, n (%)			
Primi	4898 (25.2%)	270 (25.8%)	507 (29.3%)
Multi	14,526 (74.8%)	777 (74.2%)	1226 (70.7%)
Refugee status, *n* (%)			
Refugee	14,393 (74.1%)	592 (56.5%)	707 (40.8%)
Migrant	4450 (22.9%)	362 (34.6%)	951 (54.9%)
Visitor (to refugee camp)	580 (3.0%)	93 (8.9%)	75 (4.3%)
EGA first ANC, weeks, median (range)	9 (−1–14)^a^	10 (0–14)	9 (0–14)
Anemia at first ANC, *n* (%)			
None	17,652 (95.5%)	777 (83.9%)	1569 (92.9%)
Moderate	825 (4.5%)	148 (16.0%)	120 (7.1%)
Severe	3 (0.02%)	1 (0.1%)	0 (0%)
First trimester weight, kg, mean (SD)	48.0 (7.7)	46.5 (6.1)	46.7 (6.8)
Weight gain, kg, mean (SD)	9.0 (3.9)	8.0 (3.6)	8.2 (3.6)
Chronic HTN, *n* (%)			
No	19,266 (99.2%)	1040 (99.3%)	1723 (99.4%)
Yes	158 (0.8%)	7 (0.7%)	10 (0.6%)
Number of consultations, median (range)	20 (1–43)	19 (1–37)	22 (1–38)
Year of delivery, median (range)	2007 (1986–2016)	2000 (1987–2015)	2006 (1987–2016)
EGA delivery, weeks, median (range)	39 (28–44)	39 (28–45)	39 (28–44)
Place of delivery			
Home	4920 (25.3%)	576 (55.0%)	730 (42.1%)
SMRU	12,909 (66.5%)	441 (39.3%)	815 (47.0%)
Hospital	1595 (8.2%)	60 (5.7%)	188 (10.9%)

*SD* standard deviation, *EGA* estimated gestational age, *ANC* antenatal clinic, *HTN* hypertension, *SMRU* Shoklo Malaria Research Unit (SMRU)

^a^(−1) permitted given known margins of error of this measurement

Women with malaria were more likely to be migrants, anemic, weigh less during the first trimester, gain less weight during the pregnancy, and deliver at home (Table 1). These patterns suggest that women with malaria during pregnancy had less access to health care.

### Association between falciparum malaria and hypertensive disorders of pregnancy

**Fig. 2:**
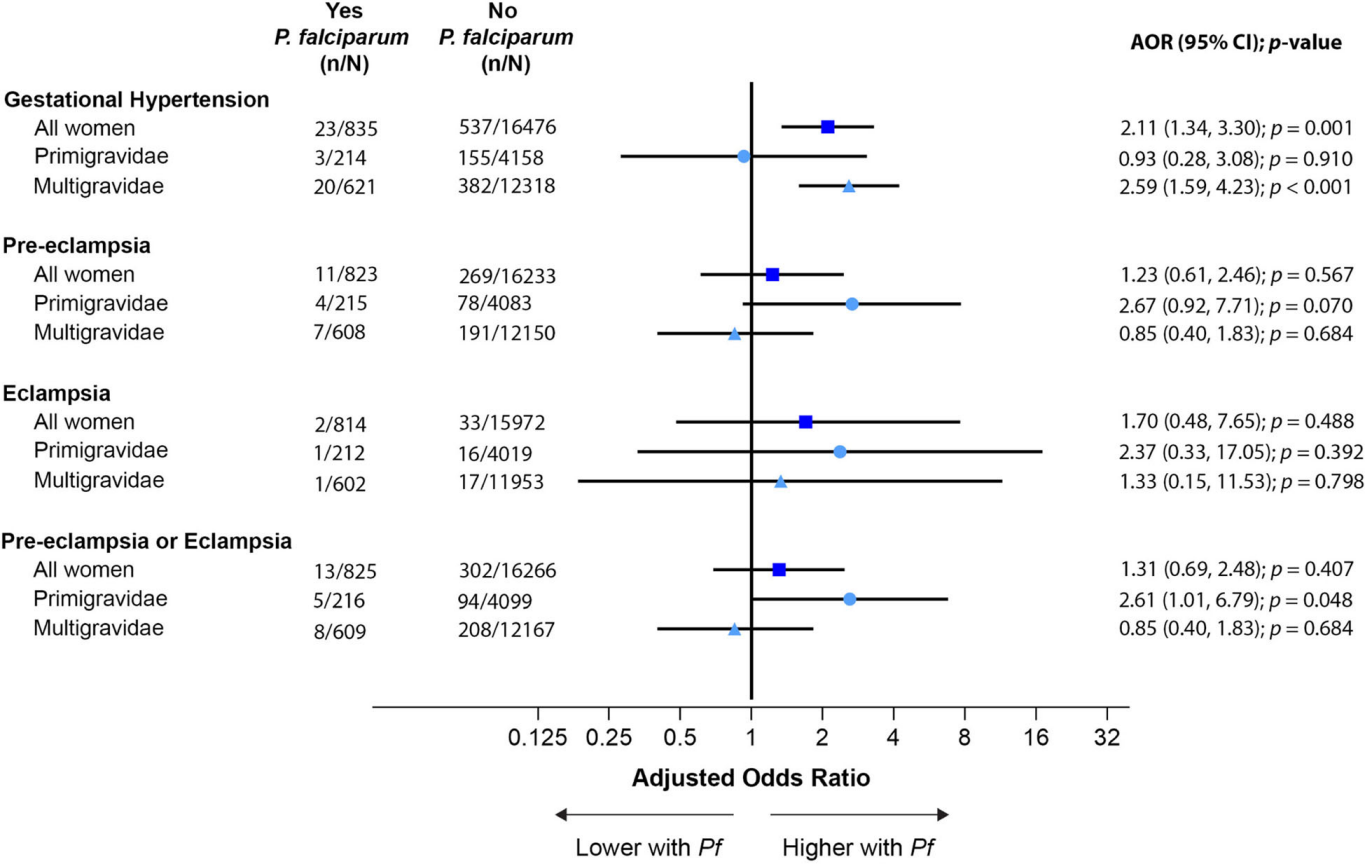
Association between falciparum malaria and hypertensive orders of pregnancy. Falciparum malaria was associated with gestational hypertension amongst all women and multigravidae, a non-significant association with pre-eclampsia amongst primigravidae, and an association with the composite outcome of pre-eclampsia/eclampsia amongst primigravidae

The overall risk of HDoP was low; most women, 21,168 (95.3%), did not become hypertensive, 620 (2.8%) had gestational hypertension, 346 (1.6%) had pre-eclampsia, and 43 (0.2%) had eclampsia. Falciparum malaria during pregnancy was associated with gestational hypertension in multigravidae (AOR 2.59, 95%CI 1.59–4.23, *p* < 0.001) but not primigravidae (Fig. 2). In contrast, amongst primigravidae but not multigravidae, there was an association with pre-eclampsia (AOR 2.67, 95%CI 0.92–7.71, *p* = 0.07) which was statistically significant when assessing the combined outcome of pre-eclampsia/eclampsia (AOR 2.61, 95%CI 1.01–6.79, *p* = 0.048) (Fig. 2). The effect was greatest amongst young (< 20 years) primigravidae (pre-eclampsia: AOR = 3.64, 95%CI 1.04–12.79, *p* = 0.044; pre-eclampsia/eclampsia: AOR 3.23, 95%CI 1.07–9.75, *p* = 0.038).

### Association between falciparum malaria and hypertensive disorders of pregnancy by gestational age of falciparum malaria

**Fig. 3:**
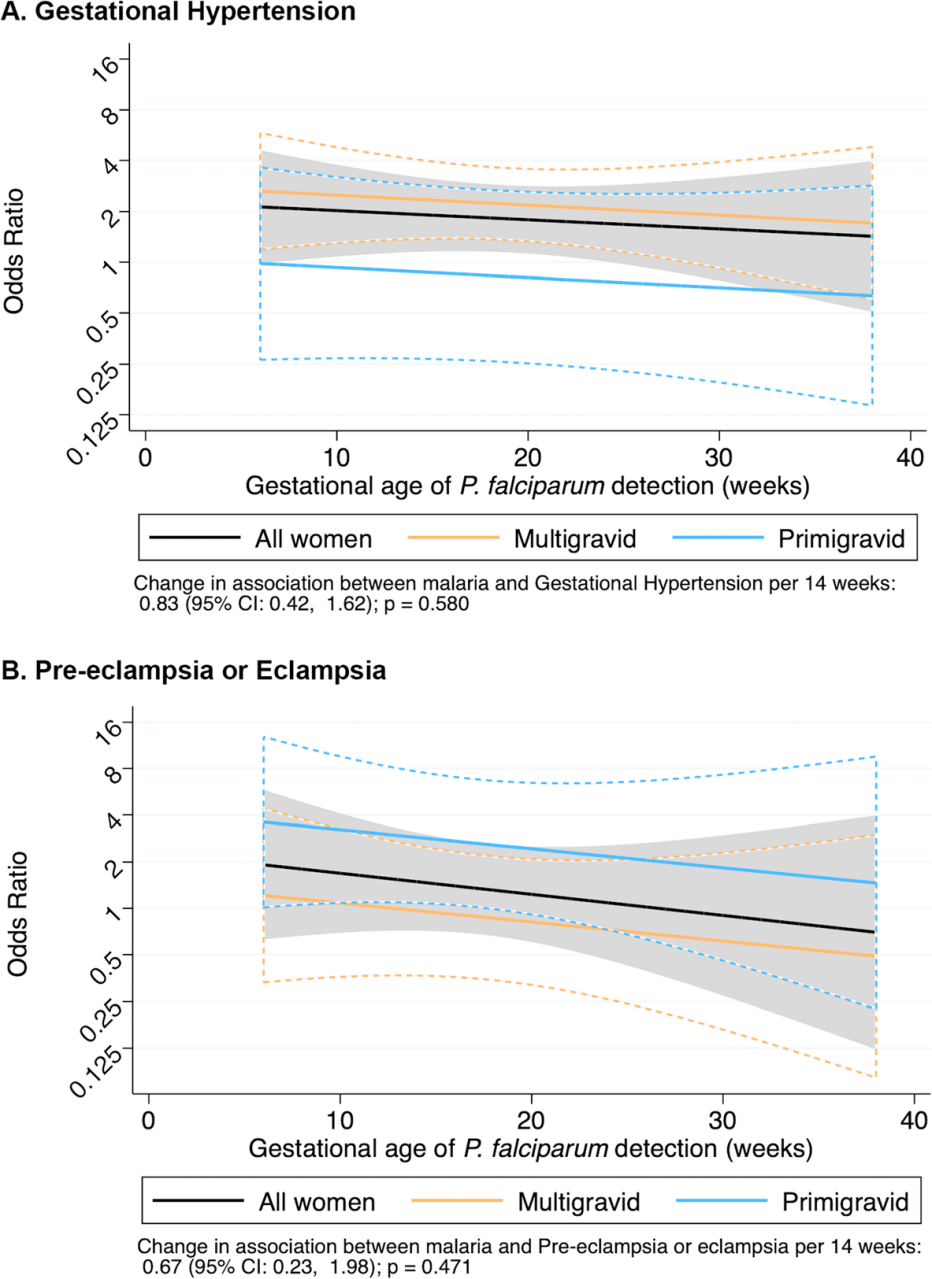
Gestational age of the first detection of falciparum malaria to predict hypertensive disorders of pregnancy. Black line: all women; blue line: primigravidae, yellow line: multigravidae. Dashed curves represent 95% confidence intervals. There was a non-significant interaction between gestational age and falciparum infection for both gestational hypertension (**a**) and pre-eclampsia/eclampsia (**b**), such that infections early in pregnancy had the greatest impact

As abnormalities in spiral artery remodeling are associated with subsequent HDoP, we specifically considered whether the effects described above varied by gestational age of the first detection of falciparum malaria. For all outcomes, there was a non-significant interaction with the gestational age of the first detection, whereby the association between falciparum malaria in pregnancy and HDoP was greater in early pregnancy than in late pregnancy (Fig. 3). When these models were used to calculate the predicted effect sizes at the beginning of the 2nd (14 weeks’ gestation) and 3rd (28 weeks’ gestation) trimesters, the associations between falciparum malaria and gestational hypertension in multigravidae and pre-eclampsia/eclampsia in primigravidae were more significant at 14 weeks (gestational hypertension amongst multigravidae: AOR 2.36, 95%CI 1.37–4.09, *p* = 0.002; pre-eclampsia/eclampsia amongst primigravidae: AOR 2.88, 95%CI 1.08–7.64, *p* = 0.034) than at 28 weeks (gestational hypertension amongst multigravidae: AOR = 1.95, 95%CI 1.01–3.77, *p* = 0.05; pre-eclampsia/eclampsia amongst primigravidae: AOR 1.94, 95%CI 0.54–6.94, *p* = 0.31) (Additional file 4).

### Association between falciparum malaria and hypertensive disorders of pregnancy restricted to women with a single episode

Because of the inverse correlation between gestational age at the first episode and the number of falciparum episodes, we performed a sensitivity analysis in which we restricted evaluation to those women who had only a single detected episode compared to those who had no detected episodes. This attenuated the relationship between falciparum malaria and gestational hypertension amongst multigravidae (AOR 1.68, 95%CI 0.87–3.27, *p* = 0.126) suggesting a dose effect. The relationship between falciparum malaria and pre-eclampsia/eclampsia amongst primigravidae was preserved (AOR 3.04, 95%CI 1.07–8.70, *p* = 0.038).

### Association between vivax malaria and hypertensive disorders of pregnancy

**Fig. 4:**
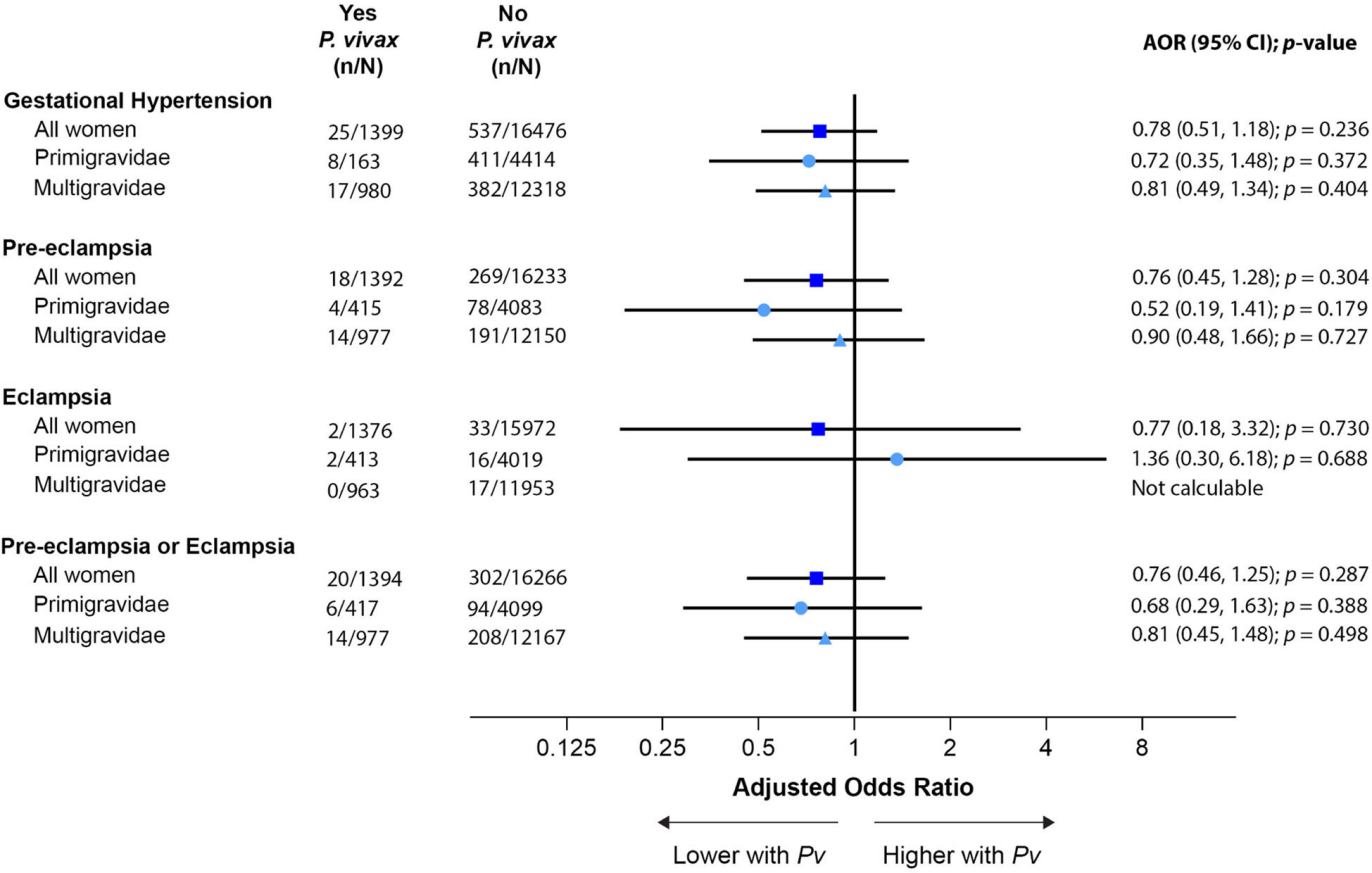
Association between vivax malaria and hypertensive disorders of pregnancy. Vivax malaria was not associated with risk of hypertensive disorders of pregnancy

**Fig. 5:**
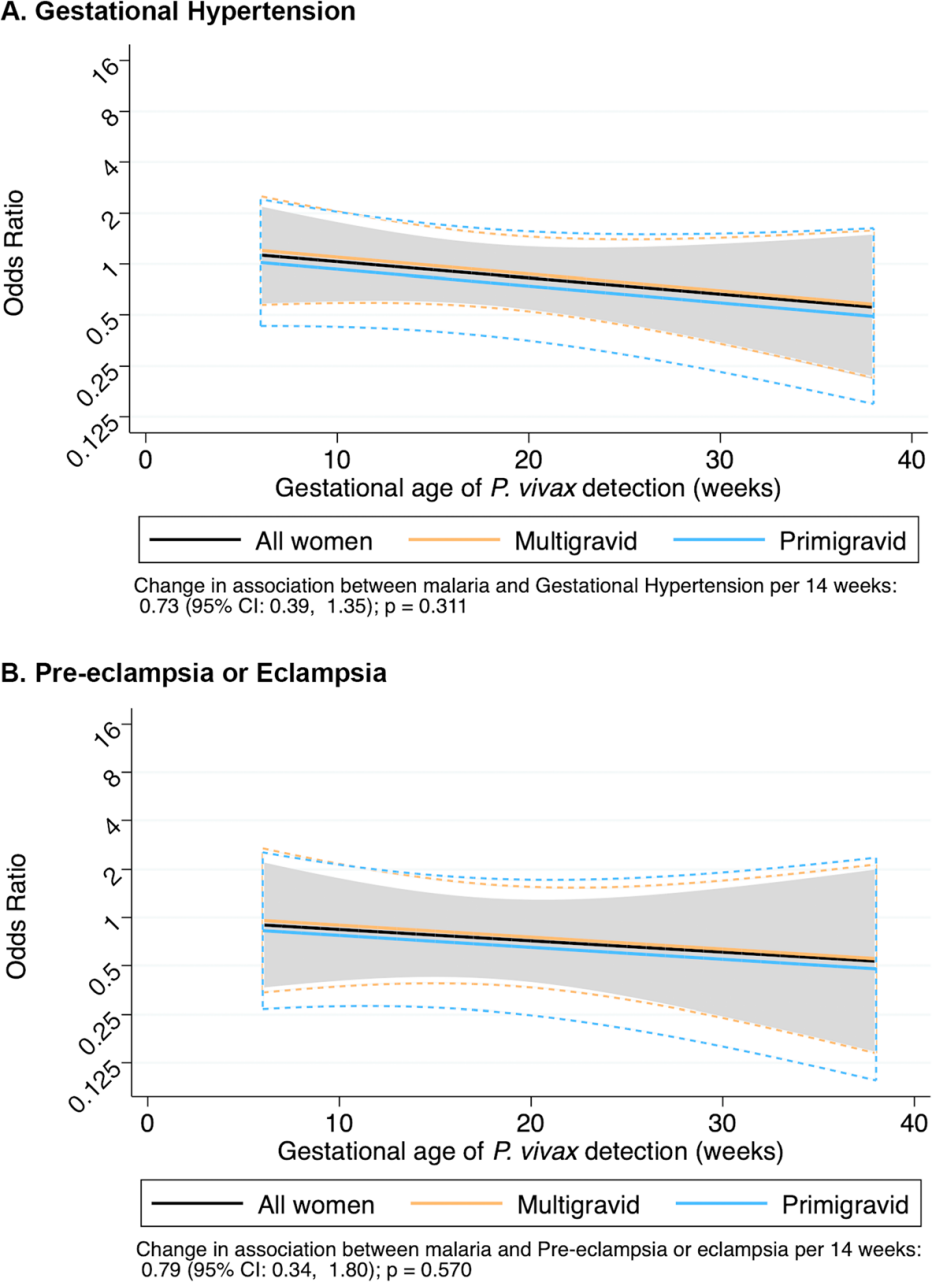
Gestational age of the first detection of vivax malaria to predict hypertensive disorders of pregnancy. Black line: all women; blue line: primigravidae, yellow line: multigravidae. Dashed curves represent 95% confidence intervals. There was a non-significant interaction between gestational age and vivax infection for both gestational hypertension (**a**) and pre-eclampsia/eclampsia (**b**). However, vivax malaria was not significantly associated with any HDoP at any point across pregnancy, and there was no evidence of effect modification by gravidity

In contrast to falciparum malaria, there was no association between vivax malaria and any outcome, nor was there any evidence of effect modification by gravidity (Figs. 4 and 5).

## Discussion

This study is the largest, prospective analysis of the relationship between falciparum and vivax malaria in pregnancy and HDoP with more than 20,000 pregnancies followed from the first trimester. It was conducted in an area of low seasonal transmission in epidemiological conditions in which symptomatic malaria in pregnancy is common and severe falciparum malaria a significant risk. We found that falciparum malaria was associated with gestational hypertension in multigravidae but not primigravidae. This effect was greater with multiple episodes. In contrast in primigravidae, there was a significant association between falciparum malaria and pre-eclampsia or eclampsia which was not seen in multigravidae. There was some indication that the effect of falciparum malaria was greater when the infection occurred early in pregnancy, but this was not conclusive. These observations are consistent with falciparum malaria impacting spiral artery development and emphasize the importance of malaria prevention early in pregnancy.

There is some evidence that systemic infections may trigger pre-eclampsia, although findings are mixed [27, 28]. Some, but not all, studies from Africa, where malaria transmission is substantially higher and most women with malaria in pregnancy are asymptomatic, have described an association between falciparum placental malaria diagnosed at delivery and HDoP [17, 21–23, 26]. In contrast, we describe the risk associated with treated peripheral falciparum malaria. In this setting, with frequent active screening and treatment infections are cleared rapidly, and there is essentially no placental malaria at delivery. However, despite prompt diagnosis and treatment, falciparum malaria early in pregnancy is associated with poor outcomes including miscarriage [12], small for gestational age [13], low birth weight [15], and pre-term delivery [15], and there is accumulating evidence suggesting that falciparum malaria early in gestation may alter placentation and spiral artery remodeling, leading to chronic placental hypoxia and alterations in the placental structure [10, 14, 29].

In this study, there was evidence of effect modification by gravidity. A previous study demonstrated an association between placental malaria and hypertension specifically in young primigravidae [21]. Primigravidae are at the greatest risk of pre-eclampsia [1] as well as falciparum malaria during pregnancy [30]. When infected, they may therefore be more susceptible to the “triggering” of a pre-eclampsia phenotype [30]. By contrast, multigravidae are more resistant to malaria infection and may be more resistant to the triggering of pre-eclampsia, ultimately leading to the milder presentation of gestational hypertension. Supporting the notion of a stronger stimulus required to trigger the phenotype amongst multigravidae, the sensitivity analysis suggested that repeat falciparum infections increased the risk of gestational hypertension. Alternatively, gestational hypertension and pre-eclampsia may be driven by different pathologic perturbations, where gestational hypertension in multigravidae may represent a disease of systemic vasculature, rather than a placental-origin disease [5].

In contrast to falciparum malaria, no increased risk of HDoP was conferred by vivax malaria. The lack of association between vivax malaria, also a systemic infection, and HDoP is striking, especially given that there were nearly twice as many episodes of vivax malaria detected relative to falciparum malaria, so the power to detect an association should have been high. In this area, vivax in pregnancy is associated with an increased risk of miscarriage, preterm birth, stillbirth, small for gestational age, and neonatal mortality [13, 16, 31]. However, vivax malaria does not result in severe multisystem disease, and although in vitro it has been shown to cytoadhere to chondroitin sulfate A [32], a placental ligand, it does not sequester significantly in the placenta or alter trophoblast invasion [33]. We hypothesize that the effect of falciparum, but not vivax, malaria on placental vascular development and function may lead to increased risk of HDoP. Alternatively, chloroquine, the treatment of vivax malaria, has inherent anti-inflammatory properties which may have been protective against the triggering of HDoP [34, 35].

This study has a number of limitations. First, as an observational cohort study over 30 years, there may be factors outside of malaria incidence [13] that varied over time, which we accounted for by adjusting for the year of delivery. In addition, we utilized the older definition of pre-eclampsia (hypertension plus proteinuria), as we had limited clinical information to define other markers of end-organ dysfunction, and clinically, this definition guided treatment. The rates of hypertensive disorders we describe are similar to those reported previously from Asia [2, 3]. Further, when considering the effect of timing of malaria episodes, we considered only the first detection. Most malaria infections occurred early in gestation, increasing the confidence intervals around the estimates of the effect in later pregnancy. In addition, similar to other developing world populations, the numbers of primigravidae relative to multigravidae were relatively small, and confidence intervals were wide. We have attempted to control for known potential confounders, but there may be other non-assessed residual confounders. Importantly, women with malaria likely had less access to care and were more likely to deliver at home, suggesting that the diagnosis of HDoP may be underestimated in this group. This, combined with prompt detection and treatment of all malaria episodes, suggests that the association between malaria and HDoP reported here is likely to be a conservative estimate of the true effect.

## Conclusions

The data from these women prospectively followed from the first trimester suggest that falciparum malaria may result in chronic placental hypoxia, eventually progressing to clinical gestational hypertension or pre-eclampsia. Worldwide, most malaria prevention strategies are not implemented until the beginning of the second trimester or later, leaving women at risk of infection early in gestation. These data highlight the critical need to prevent malaria early in pregnancy, particularly amongst primigravidae who are most vulnerable to the severe outcome of pre-eclampsia.

## Supplementary Information

**Additional file 1:** **Table S1.** *P. falciparum* to predict HDoP among women with data on body mass index from enrollment (*n*=14,590).

**Additional file 2:** **Table S2.** 1 *P. falciparum* to predict HDoP among women with known smoking status between 1997-2016 (*n*=19,049).

**Additional file 3:** **Figure S1.** First detection of plasmodium on blood smear by gestational age.

**Additional file 4:** **Table S3.** Association between falciparum malaria at 14 and 28 weeks’ gestation and HDoP.

## Data Availability

The data used to support the findings of this study may be released upon application to the Data Access Committee at Mahidol-Oxford Tropical Medicine Research Unit (MORU); contact at https://www.tropmedres.ac/units/moru-bangkok/bioethics-engagement/data-sharing.

## Abbreviations

ANC: Antenatal clinic
AOR: Adjusted odds ratio
BMI: Body mass index
BP: Blood pressure
HdoP: Hypertensive disorders of pregnancy
SMRU: Shoklo Malaria Research Unit
