# Supplementary material for: Falciparum but not vivax malaria increases the risk of hypertensive disorders of pregnancy in women followed prospectively from the first trimester
Source: BMC Med. 2021 Apr 27;19:98. doi: 10.1186/s12916-021-01960-3 (PMC8077872; doi:10.1186/s12916-021-01960-3)
Supplement: Supplementary file 1 — Additional file 1: Table S1. P. falciparum to predict HDoP among women with data on body mass index from enrollment (n=14,590). [file 12916_2021_1960_MOESM1_ESM.docx]

**Additional File 1**

**First trimester weight is a good proxy for body mass index**

Height to generate a body mass index (BMI) was available for only 14,590 women. To test the assumption that modeling first trimester weight was a reasonable surrogate for BMI, we constructed models for the subset of women for which BMI was available, including either first trimester weight or BMI to compare their relative effect. Both models included weight gain across the pregnancy as a separate parameter, as well as the other covariates detailed in the methods section. The estimates generated for all outcomes in the two models were nearly identical to each other (**Additional Table 1**). As a result, we modeled first trimester weight in the larger cohort. The estimates generated in this subset analysis, as well as the larger full cohort analysis, were consistent in all cases.

**Additional Table 1. *P. falciparum* to predict HDoP among women with data on body mass index from enrollment (n=14,590)**

| **Outcome** | | **Model with Weight** | | **Model with BMI** | |
| --- | --- | --- | --- | --- | --- |
|  |  | **AOR (95% CI)** | **p-value** | **AOR (95% CI)** | **p-value** |
| **Gestational hypertension** | |  |  |  |  |
|  | All women | 2.11 (1.11, 4.03) | 0.02 | 2.14 (1.12, 4.09) | 0.02 |
|  | Primigravidae | 1.07 (0.24, 4.74) | 0.93 | 1.12 (0.26, 4.93) | 0.88 |
|  | Multigravidae | 2.68 (1.31, 5.50) | 0.01 | 2.68 (1.32, 5.51) | 0.01 |
|  |  |  |  |  |  |
| **Pre-eclampsia** | |  |  |  |  |
|  | All women | 1.53 (0.71, 3.32) | 0.28 | 1.56 (0.73, 3.34) | 0.26 |
|  | Primigravidae | 3.42 (1.01, 11.58) | 0.05 | 3.38 (1.00, 11.38) | 0.05 |
|  | Multigravidae | 0.96 (0.44, 2.07) | 0.91 | 0.99 (0.46, 2.12) | 0.99 |
|  |  |  |  |  |  |
| **Pre-eclampsia & Eclampsia** | |  |  |  |  |
|  | All women | 1.63 (0.78, 3.40) | 0.19 | 1.65 (0.80, 3.40) | 0.18 |
|  | Primigravidae | 2.76 (0.82, 9.26) | 0.10 | 2.74 (0.82, 9.16) | 0.10 |
|  | Multigravidae | 1.22 (0.54, 2.76) | 0.63 | 1.25 (0.56, 2.78) | 0.59 |
| *Models are restricted to women with body mass index (BMI) data available from first trimester, with* | | | | |  |
| *either first trimester weight or BMI modeled. Both models include weight gain across the pregnancy* | | | | |  |
| *as a separate parameter.* | |  |  |  |  |
