# Supplementary material for: Falciparum but not vivax malaria increases the risk of hypertensive disorders of pregnancy in women followed prospectively from the first trimester
Source: BMC Med. 2021 Apr 27;19:98. doi: 10.1186/s12916-021-01960-3 (PMC8077872; doi:10.1186/s12916-021-01960-3)
Supplement: Supplementary file 2 — Additional file 2: Table S2. 1 P. falciparum to predict HDoP among women with known smoking status between 1997-2016 (n=19,049). [file 12916_2021_1960_MOESM2_ESM.docx]

**Additional File 2.**

**Smoking does not confound the association between malaria and HDoP**

To assess for possible confounding by smoking, we conducted a subgroup analysis from 1997 when collection of smoking data at first ANC visit became routine (19,049 followed from first trimester). However, because the rate of falciparum malaria fell over time [1], this restriction resulted in the loss of 42% of the women who experienced falciparum malaria (from 1,047 to 605). Among the subgroup, the effect of falciparum to predict risk of HDoP was slightly attenuated (**Additional Table 2.1**) but this appeared to be largely the effect of loss of falciparum events, rather than adjustment for smoking. Interestingly, the relationship between falciparum malaria and pre-eclampsia among primigravidae and gestational hypertension among multigravidae was largely preserved. The subgroup analysis resulted in a loss of 18% of women who experienced vivax malaria (from 1,733 to 1,350). Adjustment for smoking status did not significantly change the effect of vivax malaria (**Additional Table 2.2**).

**Additional Table 2.1 *P. falciparum* to predict HDoP among women with known smoking status between 1997-2016 (n=19,049)**

| **Outcome** | | **Adjustment for Smoking** | | | |
| --- | --- | --- | --- | --- | --- |
|  |  | **Not adjusted** | | **Adjusted** | |
|  |  |  |  |  |  |
|  |  | **AOR (95% CI)** | **p-value** | **AOR (95% CI)** | **p-value** |
| **Gestational Hypertension** | |  |  |  |  |
|  | All women | 1.73 (0.96, 3.12) | 0.07 | 1.67 (0.92, 3.02) | 0.09 |
|  | Primigravidae | 0.83 (0.19, 3.58) | 0.80 | 0.82 (0.19, 3.53) | 0.79 |
|  | Multigravidae | 2.15 (1.13, 4.09) | 0.02 | 2.06 (1.07, 3.94) | 0.03 |
|  |  |  |  |  |  |
| **Pre-eclampsia** | |  |  |  |  |
|  | All women | 1.26 (0.59, 2.70) | 0.56 | 1.18 (0.55, 2.52) | 0.67 |
|  | Primigravidae | 2.71 (0.80, 9.15) | 0.11 | 2.61 (0.78, 8.72) | 0.12 |
|  | Multigravidae | 0.80 (0.37, 1.81) | 0.57 | 0.75 (0.35, 1.62) | 0.46 |
|  |  |  |  |  |  |
| **Pre-eclampsia & eclampsia** | |  |  |  |  |
|  | All women | 1.32 (0.64, 2.70) | 0.46 | 1.23 (0.60, 2.51) | 0.58 |
|  | Primigravidae | 2.20 (0.66, 7.40) | 0.20 | 2.12 (0.64, 7.05) | 0.22 |
|  | Multigravidae | 0.99 (0.45, 2.20) | 0.98 | 0.92 (0.41, 2.03) | 0.83 |

**Additional Table 2.2. *P. vivax* to predict HDoP among women with known smoking status between 1997-2016 (n=19,049)**

| **Outcome** | | **Adjustment for Smoking** | | | |
| --- | --- | --- | --- | --- | --- |
|  |  | **Not adjusted** | | **Adjusted** | |
|  |  |  |  |  |  |
|  |  | **AOR (95% CI)** | **p-value** | **AOR (95% CI)** | **p-value** |
| **Gestational Hypertension** | |  |  |  |  |
|  | All women | 0.73 (0.46, 1.17) | 0.19 | 0.72 (0.45, 1.15) | 0.17 |
|  | Primigravidae | 0.61 (0.27, 1.40) | 0.24 | 0.61 (0.27, 1.40) | 0.24 |
|  | Multigravidae | 0.80 (0.46, 1.40) | 0.44 | 0.78 (0.45, 1.37) | 0.39 |
|  |  |  |  |  |  |
| **Pre-eclampsia** | |  |  |  |  |
|  | All women | 0.74 (0.43, 1.30) | 0.30 | 0.72 (0.41, 1.26) | 0.25 |
|  | Primigravidae | 0.49 (0.16, 1.51) | 0.21 | 0.48 (0.16, 1.50) | 0.21 |
|  | Multigravidae | 0.90 (0.47, 1.71) | 0.75 | 0.86 (0.45, 1.63) | 0.64 |
|  |  |  |  |  |  |
| **Pre-eclampsia & eclampsia** | |  |  |  |  |
|  | All women | 0.73 (0.43, 1.23) | 0.24 | 0.71 (0.42, 1.20) | 0.20 |
|  | Primigravidae | 0.53 (0.19, 1.47) | 0.22 | 0.53 (0.19, 1.47) | 0.22 |
|  | Multigravidae | 0.86 (0.46, 1.59) | 0.62 | 0.82 (0.44, 1.52) | 0.53 |

[1] Moore KA, Simpson JA, Wiladphaingern J, Min AM, Pimanpanarak M, Paw MK, Raksuansak J, Pukrittayakamee S, Fowkes FJI, White NJ et al: Influence of the number and timing of malaria episodes during pregnancy on prematurity and small-for-gestational-age in an area of low transmission. BMC Med 2017, 15(1):117.
