# Supplementary material for: Falciparum but not vivax malaria increases the risk of hypertensive disorders of pregnancy in women followed prospectively from the first trimester
Source: BMC Med. 2021 Apr 27;19:98. doi: 10.1186/s12916-021-01960-3 (PMC8077872; doi:10.1186/s12916-021-01960-3)
Supplement: Supplementary file 3 — Additional file 3: Figure S1. First detection of plasmodium on blood smear by gestational age. [file 12916_2021_1960_MOESM3_ESM.docx]

**Additional file 3.**

**Figure. First detection of plasmodium on blood smear by gestational age.**

(A) *Plasmodium falciparum*. (B) *Plasmodium vivax.* Peak detection of malaria occurred between 10 and 16 weeks gestation for both falciparum and vivax infection.

**
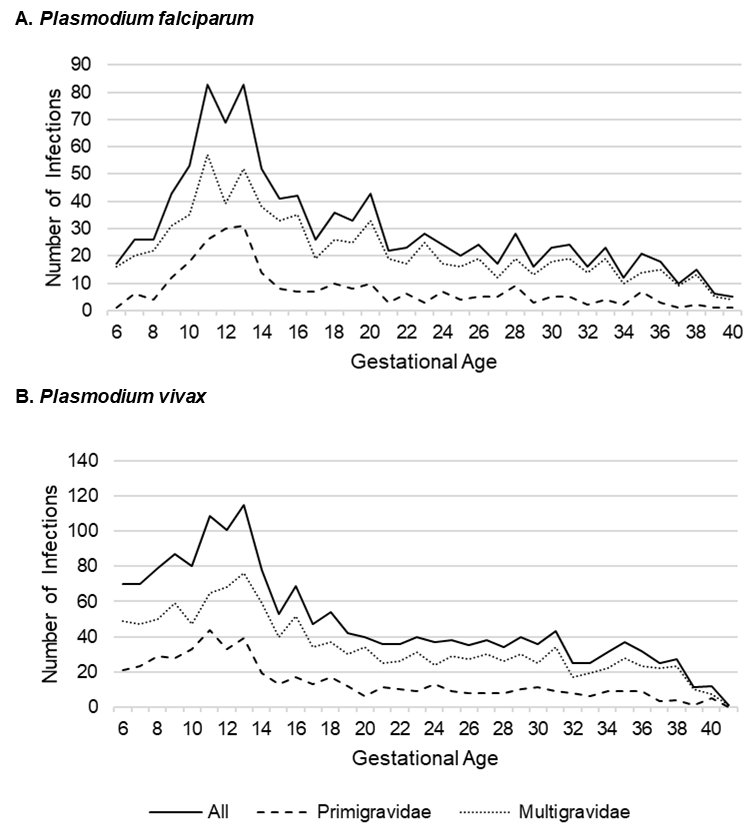
**
