# Supplementary material for: Falciparum but not vivax malaria increases the risk of hypertensive disorders of pregnancy in women followed prospectively from the first trimester
Source: BMC Med. 2021 Apr 27;19:98. doi: 10.1186/s12916-021-01960-3 (PMC8077872; doi:10.1186/s12916-021-01960-3)
Supplement: Supplementary file 4 — Additional file 4: Table S3. Association between falciparum malaria at 14 and 28 weeks’ gestation and HDoP. [file 12916_2021_1960_MOESM4_ESM.doc]

**Additional File 4**

**Table. Association between falciparum malaria at 14 and 28 weeks’ gestation and HDoP.**

| **Outcome and species** | **Gravidity** | **Gestation week of first detection** | **Predicted Association**  **(95% CI); p-value** |
| --- | --- | --- | --- |
| ***P. falciparum*** |  |  |  |
| Gestational Hypertension |  |  |  |
|  | All women | 14 | 1.92 (1.15, 3.22); p = 0.01 |
|  | All women | 28 | 1.61 (0.86, 3.04); p = 0.14 |
|  | Primigravid | 14 | 0.88 (0.27, 2.88); p = 0.83 |
|  | Primigravid | 28 | 0.73 (0.21, 2.55); p = 0.62 |
|  | Multigravid | 14 | 2.36 (1.37, 4.09); p = 0.002 |
|  | Multigravid | 28 | 1.95 (1.01, 3.77); p = 0.05 |
| Pre-eclampsia or eclampsia |  |  |  |
|  | All women | 14 | 1.49 (0.72, 3.09); p = 0.29 |
|  | All women | 28 | 0.96 (0.33, 2.79); p = 0.94 |
|  | Primigravid | 14 | 2.88 (1.08, 7.64); p = 0.03 |
|  | Primigravid | 28 | 1.94 (0.54, 6.94); p = 0.31 |
|  | Multigravid | 14 | 0.97 (0.37, 2.54); p = 0.94 |
|  | Multigravid | 28 | 0.65 (0.19, 2.18); p = 0.49 |
| ***P. vivax*** |  |  |  |
| Gestational Hypertension |  |  |  |
|  | All women | 14 | 0.95 (0.60, 1.48); p = 0.81 |
|  | All women | 28 | 0.69 (0.38, 1.29); p = 0.25 |
|  | Primigravid | 14 | 0.85 (0.41, 1.77); p = 0.67 |
|  | Primigravid | 28 | 0.62 (0.25, 1.50); p = 0.29 |
|  | Multigravid | 14 | 1.00 (0.59, 1.72); p = 0.99 |
|  | Multigravid | 28 | 0.73 (0.38, 1.41); p = 0.35 |
| Pre-eclampsia or eclampsia |  |  |  |
|  | All women | 14 | 0.79 (0.42, 1.46); p = 0.45 |
|  | All women | 28 | 0.63 (0.27, 1.44); p = 0.27 |
|  | Primigravid | 14 | 0.72 (0.28, 1.88); p = 0.50 |
|  | Primigravid | 28 | 0.57 (0.18, 1.84); p = 0.34 |
|  | Multigravid | 14 | 0.84 (0.39, 1.80); p = 0.65 |
|  | Multigravid | 28 | 0.66 (0.27, 1.63); p = 0.37 |
